# Enhancing Cellulose and Lignin Fractionation from Acacia Wood: Optimized Parameters Using a Deep Eutectic Solvent System and Solvent Recovery

**DOI:** 10.3390/molecules29153495

**Published:** 2024-07-25

**Authors:** Solange Magalhães, María José Aliaño-González, Mariana Rodrigues, Catarina Fernandes, Cátia V. T. Mendes, Maria Graça V. S. Carvalho, Luís Alves, Bruno Medronho, Maria da Graça Rasteiro

**Affiliations:** 1 University of Coimbra, CERES, Department of Chemical Engineering, Rua Sílvio Lima, Pólo II, 3030-790 Coimbra, Portugal; marianagomes.rodrigues@hotmail.com (M.R.); csfernandes@uc.pt (C.F.); catvan@eq.uc.pt (C.V.T.M.); mgc@eq.uc.pt (M.G.V.S.C.); luisalves@ci.uc.pt (L.A.); mgr@eq.uc.pt (M.d.G.R.); 2 Department of Analytical Chemistry, Faculty of Sciences, University of Cadiz, Agrifood Campus of International Excellence (ceiA3), IVAGRO, 11510 Puerto Real, Cadiz, Spain; 3 MED—Mediterranean Institute for Agriculture, Environment and Development, CHANGE—Global Change and Sustainability Institute, Faculdade de Ciências e Tecnologia, Campus de Gambelas, Universidade do Algarve, Ed. 8, 8005-139 Faro, Portugal; bfmedronho@ualg.pt; 4 FSCN, Surface and Colloid Engineering, Mid Sweden University, SE-851 70 Sundsvall, Sweden

**Keywords:** biomass fractionation, deep eutectic solvents, Box–Behnken design, acacia wood, lignocellulose

## Abstract

Cellulose and lignin, sourced from biomass, hold potential for innovative bioprocesses and biomaterials. However, traditional fractionation and purification methods often rely on harmful chemicals and high temperatures, making these processes both hazardous and costly. This study introduces a sustainable approach for fractionating acacia wood, focusing on both cellulose and lignin extraction using a deep eutectic solvent (DES) composed of choline chloride (ChCl) and levulinic acid (LA). A design of experiment was employed for the optimization of the most relevant fractionation parameters: time and temperature. In the case of the lignin, both parameters were found to be significant variables in the fractionation process (*p*-values of 0.0128 and 0.0319 for time and temperature, respectively), with a positive influence. Likewise, in the cellulose case, time and temperature also demonstrated a positive effect, with *p*-values of 0.0103 and 0.028, respectively. An optimization study was finally conducted to determine the maximum fractionation yield of lignin and cellulose. The optimized conditions were found to be 15% (*w*/*v*) of the wood sample in 1:3 ChCl:LA under a treatment temperature of 160 °C for 8 h. The developed method was validated through repeatability and intermediate precision studies, which yielded a coefficient of variation lower than 5%. The recovery and reuse of DES were successfully evaluated, revealing remarkable fractionation yields even after five cycles. This work demonstrates the feasibility of selectively extracting lignin and cellulose from woody biomass using a sustainable solvent, thus paving the way for valorization of invasive species biomass.

## 1. Introduction

The relevance of biopolymers derived from lignocellulosic biomass is on the rise in various sectors thanks to their appealing properties and promising potential to replace non-renewable-based polymers in numerous applications [1,2]. Petroleum has been the principal feedstock in various applications, including lubricants, pesticides, construction materials, plastics, polymers, surfactants, consumer products, and medicine [3,4,5]. The escalation of petroleum prices, coupled with a multitude of environmental concerns like global warming, acid rain, and glacier melting, have been increasing global awareness [6]. Thus, it is of major importance to find suitable sustainable alternatives to traditional non-renewable materials. Biopolymers derived from wood and its by-products, notably cellulose and lignin, have been emerging as reliable alternatives for replacement of some petroleum-based polymers or as sustainable raw materials for further chemical modification and tuning of their properties for specific applications [7,8,9].

Lignocellulosic biomass is a diverse and plentiful feedstock that can be obtained from a variety of sources, including hardwood, softwood, agricultural waste, and grasses [6,10,11,12]. While differing in the relative proportion of each constituent, the main compounds found in these natural resources are cellulose, hemicelluloses, lignin, and extractives. Cellulose is generally regarded as the most abundant and renewable compound found in biomass. It is a linear, partially crystalline homopolymer of glucose [13]. In the lignocellulosic biomass, cellulose is physically and chemically linked to hemicelluloses and lignin, forming a complex hierarchical matrix [14].

Lignin stands as the world’s second most abundant biopolymer, comprising approximately 15–20% of terrestrial plant biomass [15,16]. As a renewable resource, it displays several appealing characteristics, including remarkable stability, biodegradability, and antioxidant properties [17]. This biopolymer is insoluble in water and works as the “natural adhesive” that binds the cellulose fibrils within plant cells [18,19,20,21]. Moreover, lignin is recognized as a key bio-based and renewable platform of aromatic compounds due to its highly branched and amorphous structure and enriched composition of various phenol derivatives [19]. The relative proportions of the three main fundamental phenylpropane monomers, often referred to as “monolignols”, is strongly dependent on the plant type [16].

The complex molecular organization and extended network of molecular interactions of the different compounds present in the plant cell walls render their isolation and purification a challenging process [22]. Different fractionation methods have been developed; kraft, soda, and sulfite pulping are the most widely used [23,24,25]. However, these sulfur-containing processes raise important environmental concerns such as the release of sulfur dioxide into the atmosphere during, for instance, kraft [26]. Conversely, in organosolv pulping, high pressure and harsh organic solvents are typically used, increasing the costs of suitable material and safety measures [27]. In recent times, there has been a growing interest in environmentally friendly processes. Among them, the use of deep eutectic solvents (DES) to fractionate lignocellulosic materials is a highly appealing alternative [14,28].

Deep eutectic solvents (DES) are composed of a hydrogen bonding acceptor, of which one of the most commonly used is choline chloride (ChCl), and one or more hydrogen bond donors such as organic acids [14,29]. These solvents can be produced by blending various constituents in appropriate proportions to create the desired DES, which are typically characterized by a significantly reduced melting point compared to that of the individual components. DES exhibit similar characteristics to conventional ionic liquids, including low vapor pressure, selectivity, and a reduced melting point. However, they offer a more environmentally friendly profile and cost effectiveness [14]. In addition to the composition, which profoundly influences the fractionation efficiency of DES and the selectivity and purity of compounds derived from lignocellulosic biomass, several other factors also hold significant importance. Tan et al. (2020) highlighted that the extraction efficiency of acidic DES is strongly influenced by the nature of electron donors [25]. Specifically, electron donors from alkyl groups to oxygen strengthen the hydrogen bonds in the hydroxyl groups of acids. Despite this, the authors argued that a DES mixture with levulinic acid (LA) is less effective compared to those with lactic acid, acetic acid, formic acid, or glycolic acid. However, the mixture composition is not the sole factor affecting biomass fractionation; temperature and extraction time frequently exert a significant influence on the fractionation process. For instance, previous studies have demonstrated that the fractionation yield of lignin can be enhanced by employing DES at temperatures in the range of 120–140 °C. This approach has been shown to result in purer extracts [14,29]. It was also observed that the selectivity of the DES is deeply related to its composition. Upon identifying the optimal conditions, the key process variables—temperature and extraction time—were optimized to achieve maximum yields for both the cellulose and lignin fractions.

When discussing sustainability, it is essential to consider both the DES features and the overall process. In this regard, Isci et al. (2022) explored different recovery techniques for DES, including the use of water as an antisolvent [30]. Kumar et al. (2016) studied the fractionation of rice straw with choline chloride/lactic acid at 60 °C for 12 h [31]. These authors separated the solid and liquid fractions and added water to the liquid fraction to precipitate solubilized lignin. After lignin separation, the DES was successfully recycled by low-pressure evaporation at 60 °C. The recovered DES showed excellent performance with no significant loss in delignification efficiency. Similarly, Bradić et al. (2019) reported satisfactory extraction performance of lactic acid-containing DES when recycled twice, achieving an 87% chitin yield with minimal loss after recycling [32]. Satlewal et al. (2019) observed a marginal reduction in the delignification of sugarcane bagasse with recycled choline chloride acid DES, with recovery yields of 88%, 79%, and 69% after the first, second, and third recycling cycles, respectively [33]. They attributed the decline in delignification to increased impurities during recycling.

In the present work, we introduce an improved method with enhanced extraction capacity (yield and purity). Moreover, the solvent reusability (over five cycles) was evaluated to infer its recovery and efficiency (yield and purity) after repeated use. It is important to highlight that while there is extensive literature on the use of DES in biomass fractionation, little attention has been given to its recovery, recycling, and reuse. A thorough physicochemical analysis of the recovered solvents was performed to evaluate its compositional and acidity changes upon systematic recycle/reuse.

## 2. Results

### 2.1. Influence of Solvent Ratio

**Figure 1:**
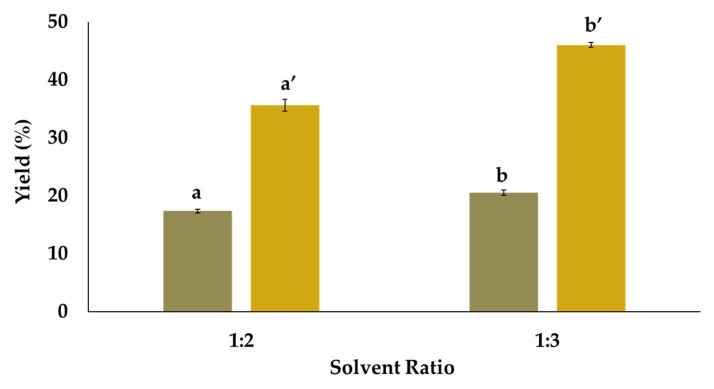
Fractionation yield of lignin (brown) and cellulose (yellow) as a function to the solvent ratio for fractionation performed at 120 °C during 1 h. Different letters above the columns imply significant differences at 95% confidence.

Initially, the influence of the solvent ratio in the fractionation of lignin and cellulose from acacia wood was evaluated with 1:2 and 1:3 molar ratios. Each test was conducted in duplicate. The lignin and cellulose contents are represented in Figure 1.

As can be observed, the 1:3 ratio allowed for the highest fractionation of lignin (i.e., 21%) and cellulose (i.e., 46%). In addition, a one-way ANOVA was performed, resulting in *p*-values of 0.017 and 0.006 for lignin and cellulose, respectively. These results demonstrate the impact of the solvent ratio on the fractionation of both compounds. The 1:3 ratio yielded the most favorable result and was selected for subsequent testing.

### 2.2. Lignin Fractionation

After selecting the most suitable solvent ratio, the time and temperature were evaluated regarding the fractionation of the lignin using a BBD-RSM. As previously alluded, this design considers nine experiments that were randomly performed. The lignin content was quantified as previously described and used as the first response variable.

The R-squared statistic indicates that the model explains 93.94% of the variability in lignin content. The Durbin–Watson (DW) statistic evaluates the residuals to determine if there is any significant correlation based on the order in which the data are supplied in the file. Since the *p*-value is higher than 5%, there is no significant autocorrelation in the residuals. The predicted and observed values were compared, obtaining an average deviation of 4.50% (ranged from 0.73% to 8.85%). The sum of squares, the F values, and the *p*-values for the two variables are presented in Table 1.

**Figure 2:**
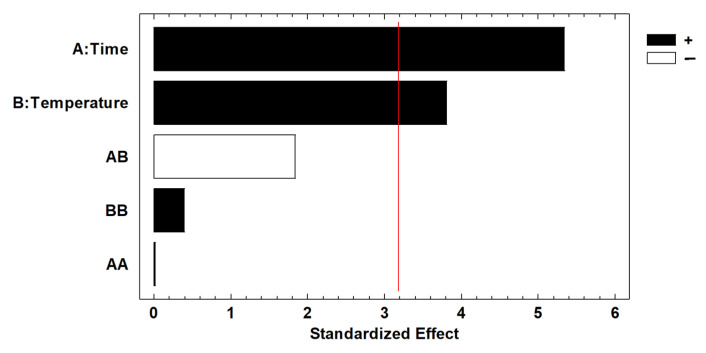
Pareto chart for lignin content after lignin fractionation using BBD-RSM. The red line represents 95% significance.

The analysis revealed that both the time and the temperature of fractionation are influential variables in the lignin content, with *p*-values of 0.013 and 0.032, respectively. The results are graphically represented in a Pareto chart (Figure 2). The influence of both variables on the response was found to be positive, indicating that the obtained lignin content was higher when the values of time and temperature were at the low extreme of the studied range. Furthermore, it can be observed that the interaction between both variables is not a significant factor.

The coefficients obtained for the second-order polynomial equation regarding the lignin content resulted in the following expression:

Y = 10.495 + 12.871·X_1_ − 0.301·X_2_ + 0.003·X_1_^2^ − 0.069·X_12_ + 0.003 · X_2_^2^ (1)

In Equation (1), Y is the lignin content (%), X_1_ is the time of fractionation, and X_2_ is the temperature of fractionation.

The optimal conditions for each variable were identified using the BBD-RSM. The optimal fractionation conditions were determined to be 160 °C and 8 h. These conditions are consistent with those previously described by other authors in other biomass feedstocks. For instance, Cassoni et al. (2022) [34] achieved the highest lignin fractionation yield from grape stalks using DES composed of lactic acid and ChCl (5:1) at 120 °C during 5 h of fractionation. Wu et al. (2021) [35] studied the fractionation of lignin from poplar wood using DES composed of lactic acid and ChCl but with different molar ratios (1:4, 1:6, 1:8, 1:10, 1:12, and 1:14) at 120 °C for 8 h. Ovejero-Pérez et al. (2020) [36] evaluated the fractionation of lignin from eucalyptus wood and demonstrated that the highest fractionation yield was achieved at 135 °C during 6 h. Zhang et al. (2022) [37] observed that performing the fractionation at 150 °C for 5 h allowed extracting the highest concentration of lignin from corncob residues. It is important to note that the quantity of the extracted lignin cannot be straightforwardly compared, as all the aforementioned studies were conducted using lignocellulosic matrices different from the acacia wood biomass used in the present work.

### 2.3. Cellulose Fractionation

The fractions rich in cellulose obtained under the conditions described in Table 1 were analyzed and the cellulose contents determined. The developed method exhibited an R-squared statistic of 94.29% and a DW with a *p*-value of 0.9046 (higher than 5%). proving there is no indication of serial autocorrelation in the residuals. The predicted and observed values were compared, and an average deviation of 5.91% was obtained (ranging from 1.89% to 12.40%). The sum of squares, the F-values, and the *p*-values for the two variables are presented in Table 2.

**Figure 3:**
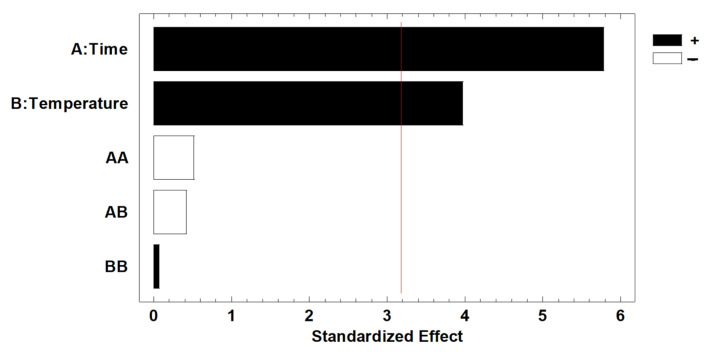
Pareto chart for cellulose content in the extracts using BBD-RSM. The red line represents 95% significance.

As observed in the case of the lignin, both parameters (time and temperature) of fractionation were found to be influential variables in the cellulose content, with *p*-values of 0.0103 and 0.0286, respectively. However, when the results are graphically represented in a Pareto chart (Figure 3), it can be observed that the influence of both variables is positive regarding the cellulose content. This indicates that the obtained cellulose content is higher when the applied time and temperature are at their higher extremes of the studied range. No influence of the interaction of both variables was detected; it was observed that the influence of both variables is positive regarding the cellulose content. This indicates that the cellulose content achieved is higher when the time and temperature applied are in the higher extremes of the range studied. No influence of the interaction of both variables was detected.

The coefficients of the second-order polynomial equation related to the cellulose content led to Equation (2):

Y = −44.78 + 11.63·X_1_ + 0.52·X_2_ − 0.25·X_1_^2^ + 0.03·X_12_ + 1.01 · 10^−3^ · X_2_^2^ (2)

In this equation, Y is the cellulose content (%), X_1_ is the time of fractionation, and X_2_ is the temperature of fractionation.

The optimal conditions for each variable were also obtained from a BBD-RSM and correspond to 160 °C and 8 h fractionation, both at the high extreme of the tested range. Therefore, it was decided to not increase these values.

As mentioned above, it is not straightforward to compare our data with the existing literature since most of the studies do not focus on the fractionation of cellulose with DES. Instead, the literature mainly focuses on fractionation using standard ionic liquids at high temperatures. Phromphithak et al. (2022) [38] evaluated the cellulose fraction obtained from corncob residues using DESs in a combination of ChCl with glycerol in the molar ratios of 1:0.5–4. The optimal conditions were determined to be 150 °C for 12 h of fractionation and for a 1:4 ChCl-to-glycerol molar ratio, in line with the time and temperature conditions obtained in the current research. Moon Lee et al. (2022) [39] also studied the cellulose fraction from oil palm empty fruit bunch using 140 °C for 30 min but applying ultrasounds. In this case, it was also demonstrated that the efficiency of incorporating ultrasounds in the pre-treatment of biomass results in a higher yield of reducing sugars in comparison to conventional heating.

### 2.4. Multi-Response Analysis

**Figure 4:**
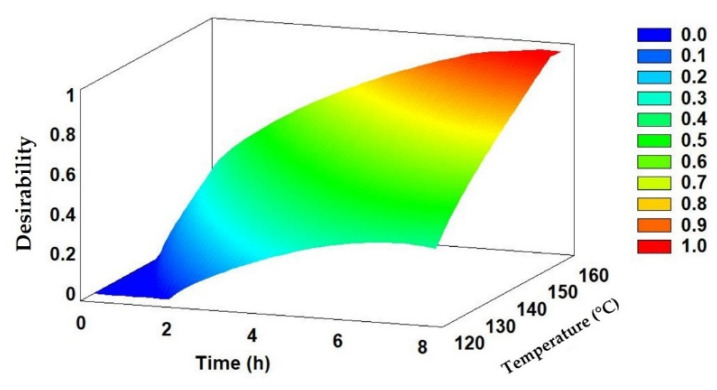
Estimated response surface graph for lignin and cellulose fractionation from acacia wood at the same time. Blue color represents the low desirability, whereas the red color represents the highest desirability.

The optimization procedure involving both variables (lignin and cellulose content) was considered in a multi-response analysis to find the best compromise between the optimization of both biopolymer yield while achieving the highest concentration of both compounds. The results are graphically represented in a response surface graph (Figure 4), which demonstrates that the highest desirability (i.e., the simultaneous highest content of lignin and cellulose) is achieved at the higher extreme of the studied range. The optimal conditions obtained were 160 °C for a fractionation performed during 8 h.

Overall, the following conditions allow the best compromise between the purity of the lignin and cellulose fractions obtained from acacia wood: 15% (*w*/*v*) of the powdered sample in 1:3 ChCl:LA in the reactor, heating the mixture in an oven at 160 °C during 8 h. As discussed, the optimal conditions for the fractionation process are at the upper limit of the range studied. Nevertheless, additional trials showed that a further increase in temperatures and time causes a significant decrease in the concentration of both compounds, most likely due to degradation of the biopolymers. For this reason, the studied range was not extended.

### 2.5. Repeatability and Intermediate Precision

The proposed combination method was validated by conducting a repeatability and intermediate precision study under optimal conditions. For repeatability, six experiments were carried out on the same day. In contrast, for the intermediate precision, three fractionations were performed on three different days. The lignin and cellulose contents were quantified, and the statistical parameter selected to assess the suitability of the selected methodology was the coefficient of variation (C.V.). The results are presented in Table 3.

It can be concluded that the developed methodology exhibits good repeatability (C.V. of 1.34% and 0.79% for lignin and cellulose, respectively) and intermediate precision (C.V. of 2.82% and 2.47% for lignin and cellulose, respectively), with C.V. lower than 5% in all cases. Therefore, the data suggest the proposed methodology is suitable.

### 2.6. Cellulose Characterization

**Figure 5:**
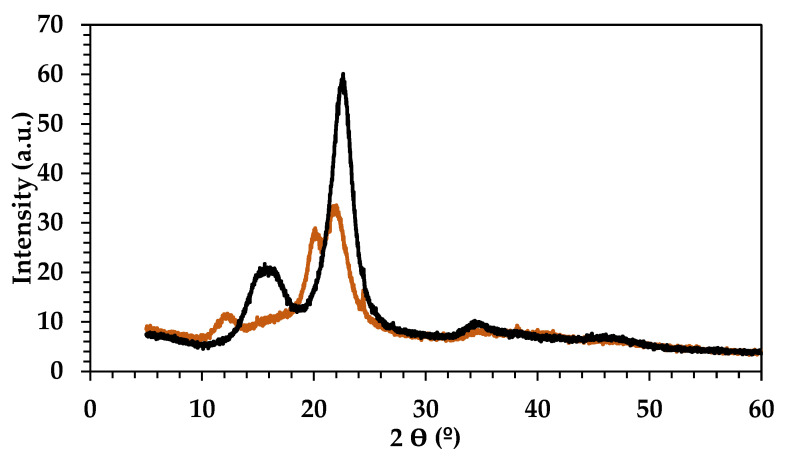
XRD diffraction pattern: smoothed raw data of cellulose extracted with DES (1:2) (black line) and cellulose extracted with DES (1:3) (orange line).

After optimizing the extraction process, the cellulose fraction was further characterized with determination of parameters such as the degree of polymerization (trough intrinsic viscosity, using CED as solvent) as well as determining its allomorph via X-ray diffraction. Because these parameters strongly depend on the extraction conditions, significant changes may indicate potential depolymerization effects and partial dissolution/regeneration processes during fractionation. Table 4 and Figure 5 show the values obtained for intrinsic viscosity, DP, and average MW, and the XRD diffractograms of the obtained celluloses.

According to the Mark–Houwink–Sakurada relationship, a lower MW implies a lower intrinsic viscosity. It was observed that the introduction of LA causes a slight decrease in cellulose MW, corroborating earlier findings [40,41]. Additionally, XRD data for the samples fractionated using 1:2 and 1:3 DES revealed relevant differences (Figure 5).

The cellulose extracted with 1:2 ChCl:LA displayed a major peak at ca. 22.5° (002), with a smaller side peak at 20.5° (021), which is typical of the cellulose I crystalline allomorph. Other characteristic reflections for cellulose I allomorph were found at 14.7° (101), 16.6° (101), and 34.7° (040) [13]. On the other hand, the cellulose sample extracted using the 1:3 ChCl:LA revealed diffraction peaks centered at 20.1 (110) and 21.9 (020) and a third reflection at 12.1 (1–10), which are assigned to the cellulose II allomorph [42]. This suggests that the solvent with a higher concentration of LA not only extracts cellulose but also induces its regeneration into a different allomorph. This change in crystalline organization suggests that cellulose is more strongly affected in the 1:3 ChCl:LA DES, possibly facilitating cellulose accessibility and/or degradation. In fact, the lower cellulose DP observed in the 1:3 ChCl:LA DES supports the enhanced dissolution of biomass in this solvent composition, accounting for the observed reduction in yield with longer fractionation times and/or higher temperatures.

### 2.7. Solvent Recovery and Reuse

As previously discussed, the optimal conditions for the fractionation of acacia wood were found to be 8 h at 160 °C, with ChCl:LA (1:3). However, it is important to note that the marginal gains between 4h and 8 h may not justify the need for higher energy consumption (in the case of 8 h extraction), particularly when carefully balancing the extraction yield and purity of both fractions. Furthermore, using the ChCl:LA (1:3) molar ratio allows obtaining cellulose II allomorph, a non-native form of cellulose. Considering all these aspects, the recovery and reuse assays focused on performing the fractionation for 4 h at 160 °C, using the ChCl:LA (1:2) DES. The objective of this approach was to not only achieve a balance between energy efficiency (lower temperature and/or less time) while maximizing the extraction yield but also to optimize the process for practical applications, targeting pristine cellulose I allomorph.

**Figure 6:**
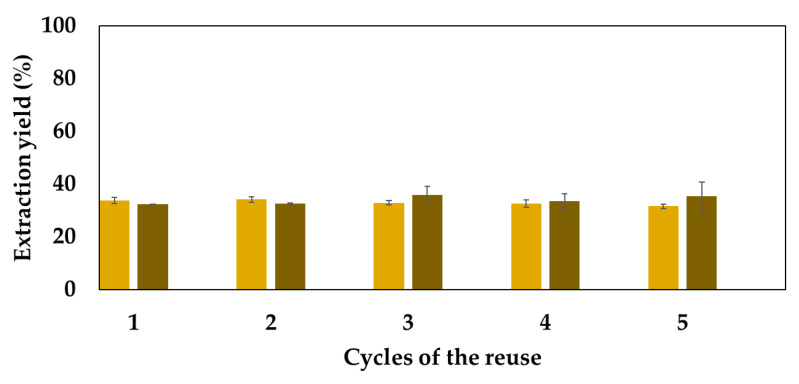
Extraction yield of both the cellulose-rich (yellow columns) and lignin-rich (brown columns) fractions during five consecutive fractionation cycles. Note that after lignin and cellulose isolation, the solvent was immediately used in a subsequent fractionation without any purification step.

As solvent reuse progressed, the yield of extraction of the cellulose-rich fraction stabilized at approximately 35–40%, while a slight yield enhancement of the lignin-rich fraction was noticed with increasing number of cycles (Figure 6). This observation suggests that the efficiency of lignin extraction improves with successive solvent reuses, potentially indicating an enhanced affinity between the solvent and lignin after each recovery cycle. Since no purification step was performed after each fractionation cycle, this phenomenon may be attributed to the cumulative concentration of various woody compounds, including acids, sugars, and other organic compounds, such as 5-hydroxymethylfurfural (5-HMF), within the solvent. The compounds possible to identify by HPLC and their concentration at each cycle are presented in Table 5.

As the solvent was reused, notable changes in composition occurred, marked by the appearance of glucose and xylose, alongside their degradation byproducts, such as 5-HMF and acetic acid. Remarkably, LA maintained a consistent concentration, ca. 100 g∙mL^−1^. Despite these fluctuations, the fractionation yield remained relatively stable during the evaluated five cycles (Figure 6). With each reuse, new compounds were extracted, while existing ones underwent hydrolysis, maintaining an equilibrium in the solvent composition and acidity levels.

**Figure 7:**
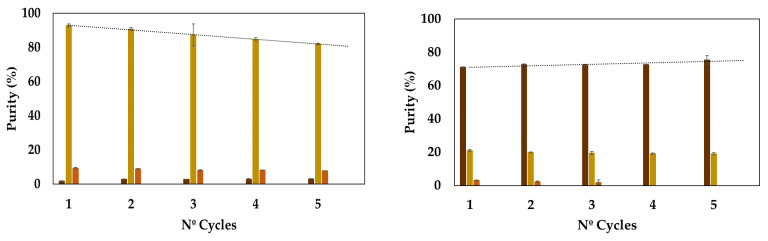
Purity of both the cellulose-rich (**left** panel) and lignin-rich (**right** panel) fractions during five cycles of acacia wood fractionation. The lignin (brown columns), cellulose (yellow columns) and hemicellulose (orange columns) contents were estimated as described in the experimental section.

Beyond assessing the extraction yield, which appeared remarkably stable throughout the five cycles evaluated, it is important to do determine whether there are any alterations in the purity of both cellulose and lignin fractions (Figure 7).

While the purity of the cellulose-rich fraction gradually declined (ca. 10%) after five consecutive recycling and reuse cycles, the lignin-rich fraction slightly increased its purity (ca. 5%). Overall, it can be concluded that this solvent’s recycling and reuse is viable. Furthermore, reuse of the solvent enhances the extraction efficacy of the lignin-rich fraction, as indicated by the rise in its acidity and the maintenance of the presence of lactic acid with the successive extractions. Francisco et al. (2012) demonstrated that lactic acid–choline chloride-based mixtures offer high lignin solubilization with poor cellulose dissolution [43]. The authors further found that a higher acid ratio improves lignin dissolution, thus supporting the current findings. Similarly, Haohe et al. (2024) demonstrated that adjusting the choline chloride/lactic acid ratio facilitates the selective extraction of lignin [44]. Additionally, their results suggested that higher levels of lactic acid boost the dissolution of cellulose.

## 3. Materials and Methods

### 3.1. Samples

The *Acacia dealbata* wood used in this work was collected in Midões (Tábua), a district of Coimbra, Portugal. The collected material was dried in an oven a 105 °C until constant weight. The branches were ground in a laboratory Wiley mill (Thomas Scientific, Swedesboro, NJ, USA) and sieved in a mechanical sieve shaker using sieves with two pore sizes (i.e., 0.25 mm and 0.84 mm) (Retsch, Serzedo—Düsseldorf, Germany). Therefore, the recovered biomass powder that was used all subsequent fractionation processes has a particle size in the range of 0.25–0.84 mm.

### 3.2. Chemicals and Solvents

Levulinic acid (98%, LA) and choline chloride (99%, ChCl) were obtained from Acros Organics (Thermo Fisher Scientific, Waltham, MA, USA). NaOH pearls were sourced from Labkem (Barcelona, Spain), and deionized water was used for preparing all solutions. H_2_SO_4_ (72% purity) from Chem-Lab (Zedelgem, Belgium) was used for lignin quantification. Intrinsic viscosity of cellulose was measured using cupriethylenediamine (CED, Panreac). For HPLC, the chemicals used were anhydrous D(+)-glucose (>99%) and D(+)-mannose (>99%) from Riedel-de Haën; acetic acid (99.8%) from Fluka; glycerol (99.5%) and L(+)-lactic acid (90%) from VWR Chemicals; and D(+)-cellobiose (98%) and 5-hydroxymethylfurfural from Acros Organics. D(+)-xylose and levulinic acid analytical standard (for HPLC) were acquired from Sigma Aldrich.

### 3.3. Preparation of DES Solvent

ChCl and LA were selected as DES components for cellulose and lignin fractionation. The selection criteria were based on their previously demonstrated suitability by some of us [14,29,36]. For both compositions, the DES was obtained by simply mixing the two compounds for 2 h at room temperature, employing a magnetic stirring apparatus [26].

### 3.4. Biomass Fractionation

#### 3.4.1. Procedure

For the fractionation of acacia wood residues, 1.5 g of the powder sample was weighted and placed in a metallic cylindric reactor. Then, 10 mL of the previously prepared DES (see Section 2.3 for details) was added to the reactor, which was carefully closed and then placed in an oven for a certain period and temperature. At the end of the fractionation, the cellulose-rich fraction (solid residue) and the lignin-rich fraction (liquid supernatant) were separated by filtration and carefully washed with deionized water until the washing waters were visually translucent. Both fractions were dried in an oven at 105 °C and kept in a desiccator. The efficiency of the fractionation was determined by quantifying the lignin and cellulose contents on both the lignin- and cellulose-rich fractions, respectively.

#### 3.4.2. Optimization of the Fractionation Process

To obtain the highest yield of cellulose and lignin from the acacia wood fractionation process, a robust optimization approach was implemented. First, the evaluation of the mixture composition (compounds ratio) in the DES was performed using a one-way ANOVA considering 95% confidence. After that, a Box–Behnken experimental design with a response surface methodology (BBD-RSM) was conducted following a 3^2^ factorial scheme to optimize the temperature and time of fractionation. This combination allows studying the influence of each variable and their interaction on the response variables, using a treatment combination of three levels per factor: (−1) a lower level, (0) an intermediate level, and (1) a higher level. Consequently, the design produced exhibits a more spherical layout devoid of axial points, which implies a reduced number of experiments and avoids the necessity of performing experiments at extreme conditions. In comparison with other designs of experiments, this represents a significant saving of resources and time. The fractionation time was evaluated at 1 h, 4.5 h, and 8 h. On the other hand, the selected fractionation temperatures were 120 °C, 140 °C, and 160 °C. These ranges were based on preliminary screening assays. When both variables were included in the design, a total of nine experiments were obtained for the optimization (Table 6). It is important to note that factorial designs with fewer than three independent variables typically do not incorporate center points, as the potential for error is less than 5%. The assays were randomly performed in duplicate, and the cellulose and lignin yields were used as the response variables for the optimization.

The estimated response value Y for the cellulose and lignin content in each test can be fitted to a polynomial equation of second degree (Equation (3)) as follows:

Y = *β*_0_ + *β*_1 × 1_ + *β*_2_X_2_ + *β*_12_X_1_X_2_ + *β*_11_X_1_^2^ + *β*_22_X_2_^2^ (3)

where Y is the response variable mentioned above, *β*_0_ corresponds to the ordinate, X_1_ (time of fractionation) and X_2_ (temperature of fractionation) are the independent variables, *β_i_* arise the linear coefficients, *β_ij_* is the coefficients of the cross products, and *β_ii_* is the quadratic coefficient.

### 3.5. Determination of the Lignin Content

LAP-004 standard procedure, established by the National Renewable Energy Laboratory (NREL), was used to assess the lignin content in the extracted fractions [19]. To do so, approximately 300 mg of the extract were weighed and subjected to hydrolysis in 3 mL solution of 12 M H_2_SO_4_ (72% (*w*/*w*)) for 60 min at 30 °C. After 1 h, the hydrolysis was allowed to continue for an additional 60 min, applying intermittent agitation. Subsequently, water was added to dilute the hydrolysates in order to reach 4% H_2_SO_4_ concentration in the solution. The solution was then autoclaved at 121 °C for 60 min and afterwards left to cool down to room temperature. Previously weighed filtering crucibles were used to vacuum filter the autoclaved solutions. The determination of “acid-insoluble lignin” was performed gravimetrically after washing the insoluble material. A UV–vis spectrometer (JASCO V650 Spectrophotometer) was used to determine the “acid-soluble lignin” by measuring the solution’s absorbance at 205 nm, after the dilution of the filtrates, to obtain meaningful absorbance values according to the following equations:

(4) $$Insoluble\;lignin\;(\%)=\frac{m_{lignin\;precipitate}}{m_{dry\;sample}}\times 100$$

(5) $$Soluble\;lignin\;\left(\%\right)=\frac{ABS\times FD\times V_{Hydrolyzed}}{\varepsilon \left(\frac{L}{g\;cm}\right)\times b\;\left(cm\right)\times m_{dry\;sample}(g)}\times 100$$

where *V_hydrolyzed_* is the volume of supernatant (soluble lignin), *m_dry sample_* is the mass of the initial sample (dry basis), *ABS* is the absorbance value of diluted supernatant at 205 nm, *FD* is the dilution factor to obtain an absorbance of supernatant at 205 nm in the range between 0.2 and 0.8, *ε* is a special extinction coefficient for lignin (110 L∙g^−1^∙cm^−1^), and *b* is the optical path length (1 cm).

### 3.6. Determination of Cellulose Content

The cellulose-rich fractions underwent hydrolysis following the procedure described for lignin determination, in accordance with the National Renewable Energy Laboratory (NREL) protocol. The samples were later characterized using high-performance liquid chromatography (HPLC). The hydrolyzed samples were filtered through a 0.2 µm syringe filter and analyzed for monosaccharides content, using an HPLC equipment (KNAUER AZURA^®^ liquid chromatography instruments, Berlin, Germany) equipped with a refractive index (RI) detector. A Rezex ROA-Organic acid H+ column (300 × 7.8 mm) maintained at 40 °C, with the corresponding guard column (at room temperature), was used. A 20 μL sample injection and a mobile phase (filtered beforehand with a 0.2 μm nylon membrane filter (Fioroni)) consisting of a 0.0025N H_2_SO_4_ aqueous solution at a flow rate of 0.6 mL·min^−1^ were the conditions used in all HPLC assays to quantify of the cellulose content in each sample. The recovered solvents were characterized for the content of carbohydrates, organic acids, alcohols, and carbohydrate degradation products, which were identified and quantified using the same setup used for cellulose quantification. The levels of carbohydrates, organic acids, alcohols, and carbohydrate degradation products in the extracted residues were identified and quantified based in the calibration curves prepared from the respective standards for HPLC.

### 3.7. Statistical Analysis

A *t*-test, with a confidence level of 95%, was performed to calculate the *p*-values of each the variables studied. The variables that presented *p*-values less than 0.05 were considered significantly influential. The ANOVA and the BBD-RSM design were generated and analyzed using the statistical software Statgraphic Centurion (version XVII) (Statgraphics Technologies, Inc., The Plains, VA, USA).

### 3.8. Viscosity Average Molecular Weight of Cellulose

The intrinsic viscosity ([η]) of all samples was determined using a standard capillary viscometer and 0.5 M CED solution at 25 ± 0.1 °C. To derive the average degree of polymerization (DP), the obtained intrinsic viscosities were converted using the Mark–Houwink equation (Equation (6)), with parameters defined by Henriksson et al. (2008) [45]. Specifically, for a DP < 950, K = 0.42 and a = 1 were employed, while for a DP > 950, K = 2.28 and a = 0.76 were used [46].

(6) $$\left[\eta \right]=K{DP}^{\alpha }$$

Treating the samples as 100% cellulose, the average molecular weight (MW) was calculated by multiplying the DP by the molecular weight of an anhydroglucose unit (AGU) (162 g∙mol^−1^).

### 3.9. X-ray Diffraction

X-ray diffraction (XRD) experiments were performed using a Siemens D5000 X-ray diffractometer. A CuKα1 radiation source with a wavelength of 1.54056 Å was used, focused by a primary Ge crystal monochromator. The detection was carried out with a standard scintillation counter, and the Cu tube operated at 40 mA and 40 kV. For precise measurements, the diffractometer featured a slit arrangement with a 2 mm pre-sample slit, a 2 mm post-sample slit, and a 0.2 mm detector slit [13,47].

## 4. Conclusions

In this work, we successfully demonstrate the efficacy of a DES system composed of ChCl:LA for simultaneous lignin and cellulose fractionation from acacia wood residues. This was achieved through the development of a statistical model based on BBD-RSM, which was optimized to achieve the highest desirability (i.e., the simultaneous maximization of lignin and cellulose contents in their respective fractions with high purity). The optimum conditions obtained correspond to the highest values for temperature and time in the studied range for the ChCl:LA (1:2) DES system. The characterization of the cellulose-rich and lignin-rich fractions revealed that the DES solvent not only excels at biomass fractionation but has also the capability to dissolve and regenerate cellulose. This versatility allows the production of high-purity cellulose in two distinct forms, namely the pristine cellulose I type and a cellulose II allomorph, by simply varying the DES ratio.

Another significant finding was the robust recyclability and reusability of the DES. Even after five consecutive cycles of recovery and reuse, the DES maintained its remarkable fractionation efficiency for both cellulose and lignin. Each successive fractionation enriched the solvent with compounds resulting from wood hydrolysis, such as different alcohols, acids, and sugars. Although this enrichment preserved the solvent acidity and extraction efficiency, thus contributing to effectively isolating lignin and hemicellulose while producing highly pure cellulose, the exact role of each degradation compound and/or other extractives in the solvent is yet to revealed. Their action mechanism regarding lignin and cellulose selective isolation will be further evaluated in future studies. This work successfully introduces a novel route to selectively extract cellulose and lignin fraction with reasonable high yield and purity. Overall, the solvent introduced presents significant advantageous features in comparison with other approaches, such as possessing no environmental risks, requiring minor efforts in its preparation, and its tunability, which allows extracting cellulose with different crystalline organization. Moreover, the recycling and reuse of the solvent (without cost-effective or time-consuming purification steps) is feasible and does not compromise the efficiency of fractionation and/or purification of recovered biopolymers.

## Figures and Tables

**Table 1:** Analysis of variance (ANOVA) of the fitted second-order polynomial model for lignin fractionation.

Variable	Sum of Squares	F-Value	*p*-Value
Time (A)	783.184	28.52	0.013
Temperature (B)	397.720	14.49	0.032
AA	0.004	0.00	0.991
AB	92.929	3.38	0.163
BB	4.2341	0.15	0.721
Total error	82.369		

**Table 2:** Analysis of variance (ANOVA) of the fitted second-order polynomial model for cellulose fractionation.

Variable	Sum of Squares	F-Value	*p*-Value
Time (A)	2443.63	33.38	0.0103
Temperature (B)	1153.47	15.75	0.0286
AA	19.4652	0.27	0.6417
AB	12.7969	0.17	0.7040
BB	0.329532	0.00	0.9507
Total error	73.2168		

**Table 3:** Results for repeatability (*n* = 6) and intermediate precision (*n* = 3 + 3 + 3) of the methodology developed.

	Repeatability	Intermediate Precision
Average lignin content (%)	68.32	68.63
Standard deviation	0.91	1.93
Coefficient of variation (%)	1.34	2.82
Average cellulose content (%)	103.42	105.85
Standard deviation	0.83	2.61
Coefficient of variation (%)	0.79	2.47

**Table 4:** Intrinsic viscosity [η] and MW of the cellulose fractions obtained after acacia wood fractionation with different DES (160 °C for 4 h).

	ChCl:LA (1:2)	ChCl:LA (1:3)
η (mL∙g^−1^)	434 (±29.04)	345 (±3.96)
DP	1034	821
Cellulose MW_iv_ (g∙mol^−1^)	167498	133060

**Table 5:** Identified secondary compounds by HPLC-RI and their estimated content in the recovered solvent after each fractionation cycle.

Compound	Concentration (g∙mL^−1^) at Each Cycle						R^2^	Equation *
	Initial	1st	2nd	3rd	4th	5th		
Glucose	0.00	0.19	0.35	0.50	0.83	1.14	1.000	Y = 1318.28·X
Xylose	0.00	0.16	0.27	0.31	0.63	0.64	1.000	Y = 1418.73·X
Lactic acid	0.00	0.00	0.00	0.28	0.40	0.80	0.999	Y = 584.80·X
Acetic acid	0.00	0.05	0.11	0.09	0.14	0.15	0.999	Y = 638.99·X
LA	116.12	112.19	98.40	93.26	97.48	102.92	0.999	Y = 1035.88·X
5-HMF	0.00	0.03	0.03	0.14	0.41	0.48	1.000	Y = 1707.11·X

* X, concentration (mg∙mL^−1^); Y, peak area.

**Table 6:** Experimental fractionation conditions for 1:3 (ChCl/LA) molar ratio and values of cellulose and lignin observed and adjusted by the BBD-RSM with two variables.

Experiment	Time (h)	Temperature (°C)	Lignin Content Observed (%)	Lignin Content Adjusted (%)	Error (%)	Cellulose Content Observed (%)	Cellulose Content Adjusted (%)	Error (%)
1	1	160	53.96 ± 0.23	57.22	6.05	69.29 ± 0.46	72.35	4.42
2	4.5	160	69.48 ± 0.72	63.78	8.20	102.63 ± 3.32	93.86	8.55
3	8	160	68.00 ± 0.02	70.43	3.58	103.42 ± 0.83	109.13	5.52
4	8	120	64.85 ± 0.21	63.79	1.63	87.30 ± 1.99	84.98	2.66
5	4.5	120	46.21 ± 0.49	47.50	2.79	58.83 ± 1.64	66.13	12.40
6	8	140	67.03 ± 0.21	65.66	2.05	100.04 ± 0.62	96.65	3.39
7	1	120	31.58 ± 0.49	31.30	0.73	46.01 ± 0.42	41.04	10.81
8	1	140	45.84 ± 0.47	42.81	6.62	54.37 ± 0.44	56.29	3.52
9	4.5	140	49.78 ± 0.44	54.19	8.85	78.11 ± 1.20	79.59	1.89

## Data Availability

Data are contained within the article.
